# Pre-hospital delay in Vietnamese patients hospitalized with a first acute myocardial infarction: A short report

**DOI:** 10.12688/f1000research.6943.3

**Published:** 2018-01-15

**Authors:** Hoa L. Nguyen, Dat T. Phan, Duc A. Ha, Quang N. Nguyen, Robert J. Goldberg

**Affiliations:** 1 Department of Quantitative Sciences , Baylor Scott & White Health, Dallas, Texas, USA; 2 Institute of Population, Health and Development, Ha Noi, Vietnam; 3 Viet Nam National Heart Institute, Ha Noi, Vietnam; 4 Ministry of Health, Ha Noi, Vietnam; 5 Department of Quantitative Health Sciences, University of Massachusetts Medical School, Worcester, Massachusetts, USA

**Keywords:** Acute myocardial infarction, pre-hospital delay, outcomes, epidemiology, Vietnam

## Abstract

**Background**: Administration of coronary reperfusion therapy to patients with an acute myocardial infarction (AMI) within the proper timeframe is essential in avoiding clinical complications and death. However, the extent of pre-hospital delay is unexplored in Vietnam. This report aims to describe the duration of pre-hospital delay of Hanoi residents hospitalized with a first AMI at the Vietnam National Heart Institute .

**Methods**: A total of 103 Hanoi residents hospitalized at the largest tertiary care medical center in the city for first AMI, who have information on  prehospital delay was included in this report.

**Results**: One third of the study sample was women and mean age was 66 years. The mean and median pre-hospital delay duration were 14.9 hours and 4.8 hours, respectively. The proportion of patients who delayed <6 , 6-<12, and ≥ 12 hours were 45%, 13%, and 42%, respectively.

**Conclusions**: Our data shows that a prolonged pre-hospital delay is often observed in patients with a first AMI in Vietnam. In order to confirm these preliminary descriptive findings, a full-scale investigation of all Hanoi residents hospitalized with first AMI is needed. Increasing public awareness about AMI treatment is vital in encouraging patients to seek medical care timely after experiencing AMI symptoms such that received treatment is most effective.

## Introduction

Vietnam is a low-middle income country, and has been in the midst of an important epidemiological transition. Over the past two decades, the overall morbidity and mortality rates resulting from non-communicable diseases (NCDs) has been rising rapidly and presents itself as a major problem in Vietnam. One quarter of all deaths annually in Vietnam are now the result of cardiovascular diseases, making it the leading cause of death.

Timely administration of reperfusion therapy to patients with an evolving acute myocardial infarction (AMI) is of utmost importance in reducing clinical complications and death. Previous research studies have convincingly proved that reperfusion treatment is most effective if patients with ST-segment elevation myocardial infarction (STEMI) are treated in a timely fashion, particularly within one hour of acute symptom onset ^1, 2^; the relation between extent of pre-hospital delay and outcomes after non-STEMI has not been firmly established.

Despite the importance of prolonged pre-hospital delay on the timely receipt of effective treatments and short-term outcomes, so far we have found no evidence of studies conducted to examine the extent of pre-hospital delay among adult patients hospitalized with AMI in Vietnam. This short report hence aims to describe the extent of pre-hospital delay in Hanoi residents who were non-transferred, hospitalized with a first AMI at the Vietnam National Heart Institute in 2010.

## Methods

### Ethical statement

This study was approved by the Institutional Review Board at the Institute of Population, Health and Development. A waiver of patient consent was approved by the Institutional Review Board. All patients’ personal information was de-identified before analysis.

### Study setting

This study was conducted at the Vietnam National Heart Institute (VNHI) ^3, 4^. This hospital is a 250 bed tertiary care medical center in Hanoi (2009 census = 6.5 million) which manages the majority of Hanoi residents hospitalized with acute coronary disease and other NCDs.

### Case ascertainment

Computerized printouts of discharged patients from the VNHI in 2010 with possible AMI were collected and International Classification of Disease codes for possible AMI (I20–I25) were reviewed. Two cardiologists validated these cases utilizing predefined criteria developed by the World Health Organization. They include having a suggestive medical history, serum enzyme increasing above the hospital’s reference range, and serial electrocardiographic (ECG) findings during hospitalization consistent with the presence of AMI; at least two of these three criteria needed to be present for an AMI to have occurred ^5^. There were 315 Hanoi residents, who satisfied the criteria for AMI were identified. However, 13 (4%) patients with history of AMI and 199 (66%) patients transferred from other facilities were excluded from the present analysis. Patients were then further divided into groups of STEMI and non-STEMI, utilizing standard classification techniques ^6^. All ECGs of potential AMI patients were reviewed by a physician under the supervision of a senior cardiologist. Due to reviewed ECGs’ inadequate quality, nine patients with AMI could not be classified as STEMI or non-STEMI.

### Data collection

For all AMI-validated cases through the aforementioned process of independent adjudication utilizing eligibility criteria, information on social demographics, clinical, medical management and hospital discharge status were collected from medical record reviews and recorded onto a standardized case report form by trained study physicians.

Pre-hospital delay is the primary outcome investigated by this study. The delay time was defined as the time interval between the onset of symptoms suggestive of AMI and the patient’s arrival at medical centers ^7, 8^. Our trained physicians reviewed any information they could seek in medical records, which described the duration of pre-hospital delay from emergency personnel, nurses, and physicians notes. Information on pre-hospital delay was collected in minutes (as a continuous variable) then was further categorized according to cut-points of 6 and 12 hours, which were commonly applied in prior studies, and based on the data distribution Patients, in whom no exact time of symptom onset was reported, and only had a delay time interval recorded in their medical record (e.g. <6, 6-<12, and ≥12 hours), were incorporated into our analysis of delay time when it was constructed as a categorical outcome. We restricted our patient population to those with information available on prehospital delay (either exact time noted or time interval) in their hospital medical records.

### Data analysis

The overall mean (standard deviation-SD) and median (inter quartile range-IQR) duration of pre-hospital delay was calculated according to standard methods. Data were shown as percentages for categorical variables and compared between patients who delayed ≥ 6 hours, and patients who sought medical care earlier, using chi-square or Fisher exact tests; medians (inter quartile range-IQR) for continuous variables were calculated and compared using the Wilcoxon sum rank tests. Since the sample size in this study was small, no regression analysis was performed. All analyses were performed using STATA 11.0 (StataCorp. TX).

## Results

A total of 103 non-transferred Hanoi residents hospitalized with a first AMI at VNHI in 2010, and had information available on prehospital delay recorded in their hospital charts were included in the report. The study sample had an average age of 66 years with a standard deviation of 13 years. One third of the study sample was women, and 69% were classified as STEMI.

### Extent of pre-hospital delay

Information on pre-hospital delay was collected in minutes (as a continuous variable) for 63% (n=65) of patients. In 37% of patients (n=38), an exact time of symptom onset was not reported, and only a delay time interval was recorded in patient’s medical record (e.g. <6, 6-<12, and ≥12 hours); these patients were included in the analysis of delay time when it was constructed as a categorical outcome.

**Figure 1.**
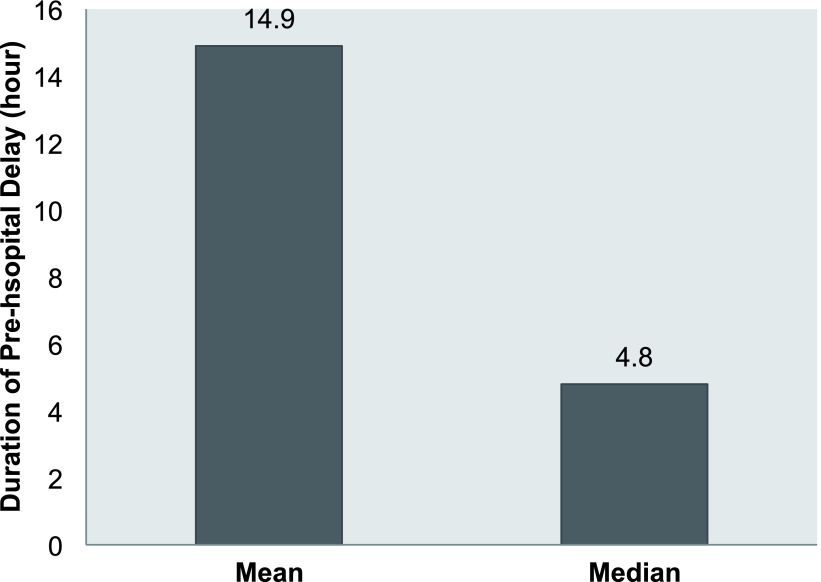
Mean and Median of Pre-hospital Delay in Patients Hospitalized with an initial Acute Myocardial Infarction.

Analysis of prehospital delay as a continuous outcome. Within the group of 65 patients reporting an exact time of symptom onset, the overall mean and median durations of pre-hospital delay were 14.9 hours (SD: 23 hours) and 4.8 hours (IQR: 3–10 hours), respectively ( Figure 1). Among patients with STEMI (n=42), mean and median delay times were 16.4 hours (SD: 26 hours) and 4.8 hours (IQR: 3.0–6.2 hours), respectively. Among patients with non-STEMI (n=19), mean and median delay times were 14.0 hours (SD: 19.2 hours) and 8.3 hours (IQR: 2.5 -12.3 hours), respectively.

**Figure 2.**
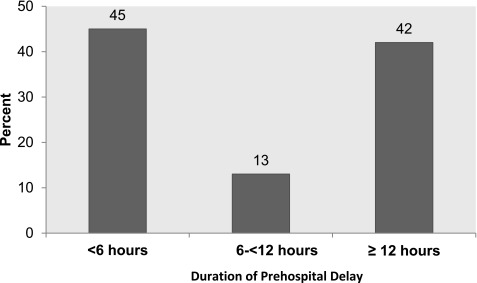
Distribution of Duration of Pre-hospital Delay in Patients Hospitalized with an initial Acute Myocardial Infarction.

Analysis of prehospital delay as a categorical outcome. When combining data from these patients (n=65), with more non-specific data from patients who reported duration of delay in a time interval (n=38), the proportion of patients who delayed <6 hours, 6-<12 hours, and ≥12 hours were 45%, 13%, and 42%, respectively ( Figure 2). Among patients with STEMI (n=65), these proportions were 51%, 12% and 37%, respectively. Among patients with non-STEMI (n=29), these proportions were 31%, 17% and 52%, respectively.

### Patient characteristics associated with prolonged pre-Hospital delay

Patients who had a delay time over 6 hours were more likely to be female compared to their counterparts, who had a delay time of less than 6 hours ( Table 1).

**Table 1.** Characteristics of Patients with an Initial Acute Myocardial Infarction (AMI) according to Extent of Delay in Seeking Medical Care.

Characteristic	< 6 hours (n=46)	≥ 6 hours (n=57)	P-value
Age (mean, SD), years	65 (13)	67 (12)	0.51
Age (n,%), years			
<60	16(34.8)	17(29.8)	0.86
60-69	11(23.9)	14(24.6)	
≥70	19(41.3)	26(45.6)	
Female (%)	11(23.9)	25(43.9)	**0.04**
Ethnicity (n,%)			
Kinh ^*^	44(95.7)	56(98.2)	0.44
Minority	2(4.3)	1(1.8)	
Have medical insurance (n,%)	22(47.8)	26(45.6)	0.82
Medical history (n,%)			
Atrial fibrillation	1(2.2)	0(0)	0.45
Coronary heart disease	5(10.9)	0(0)	NA
Diabetes mellitus	8(17.4)	11(19.3)	0.80
Heart failure	0(0)	0(0)	NA
Hypertension	30(65.2)	34(59.6)	0.56
Hyperlipidemia	0(0)	3(5.3)	NA
Stroke	5(10.9)	5(8.8)	0.72
AMI characteristics (n,%) ^†^			
STEMI	33(78.6)	32(61.5)	0.08
Non-STEMI	9(21.4)	20(38.5)	
Acute symptoms (n,%)			
Chest pain	43(93.5)	49(86.0)	0.22
Shortness of breath	21(45.7)	21(36.8)	0.37
Nausea	5(10.9)	1(1.8)	0.06
Diaphoresis	9(19.6)	12(21.1)	0.85
Fatigue	5(10.9)	7(12.3)	0.82
Clinical presentation (median, IQR)			
Heart rate, beats/min	83(67-96)	82(67-100)	0.84
Systolic blood pressure, mmHg	120(110-140)	120(110-140)	0.46
Diastolic blood pressure, mmHg	80(70-80)	80(70-90)	0.64
Laboratory findings (median, IQR)			
Total cholesterol, mmol/L	4.5(3.9-5.0)	4.6(4.0-5.2)	0.39
LDL cholesterol, mmol/L	2.5(2.3-3.1)	2.7(2.3-3.0)	0.49
eGFR (ml/min/1.73m ^2^)	71(57-79)	68(56-89)	0.56

Pre-hospital delay was defined as the duration from onset of acute symptoms suggestive of AMI to hospital arrival. P- values from chi square or Fisher exact tests for categorical variables, and t-tests or Wilcoxon-sum rank tests for continuous variables. SD: Standard deviation; IQR: Inter quartile range; STEMI: ST-segment elevation MI; LDL: Low-density lipoprotein; eGFR: estimated glomerular filtration rate ^*^The Kinh people are the majority ethnic group in Vietnam, comprising 87% of the population (census 2009). ^†^Missing data in 9 patients

Raw dataset for Nguyen *et al.*, 2015 ‘Pre-hospital delay in Vietnamese patients hospitalized with a first acute myocardial Infarction: A short report’ For all AMI-validated cases, information on social demographics, clinical, medical management and hospital discharge status were collected from medical record reviews and recorded onto a standardized case report form by trained study physicians. GFR: Glomerular filtration rate; STEMI: ST elevated myocardial infarction; LDL: Low density lipoprotein. Click here for additional data file. Copyright: © 2018 Nguyen HL et al. 2018 Data associated with the article are available under the terms of the Creative Commons Zero "No rights reserved" data waiver (CC0 1.0 Public domain dedication).

## Discussion

The results of this study indicated that Vietnamese patients experienced a considerable pre-hospital delay after the onset of AMI symptoms (median: 4.8 hours), with more than half experiencing a delay time of 6 hours or longer. Women delayed seeking care to a greater extent than men.

This report is, to our knowledge, the first in Vietnam to describe the duration of pre-hospital delays in patients hospitalized with a first AMI. In this study, patients experienced considerably longer pre-hospital delay compared with patients from both low-middle income countries and high-income countries ^7– 16^. For example, a survey of 250 patients hospitalized with AMI in Shanghai, China in 2010 reported that the median time for patients to seek treatment was around 2 hours alongside another one half hour (median time) for transportation ^9^. Another recent survey from 270 patients hospitalized with AMI in Bandar Abbas, Iran found that approximately 20% of patients experienced a delay time of 6 hours or more ^13^. A study of more than 200 patients hospitalized with acute coronary syndrome in London, England between 2001 and 2004 reported that the median pre-hospital delay time was 2 hours ^15^. Prior studies in the U.S. have shown that patients hospitalized with AMI delayed, on average, approximately 2 hours following the onset of symptoms suggestive of AMI ^11, 16^. However, the duration of pre-hospital delay in our study was comparable to that reported from more than 60 African American patients hospitalized with AMI in the US in 2003–2004 (median: 4.3 hours) ^17^ and from more than 400 patients hospitalized with AMI in Croatia in 2005 (median: 4.3 hours) ^18^. We found that comparatively, female patients tended to seek treatment much later than male patients after the onset of suggestive AMI symptoms. This result is consistent with studies conducted previously ^9, 10, 19– 22^, but contradictory to others, which reported that there was no association between sex and pre-hospital delay ^12– 14^.

Our findings emphasize the need to implement intervention programs to increase awareness of the general population with regards to the importance of seeking treatment timely after experiencing AMI suggestive symptoms. Prolonged delay may be associated with an individual’s risk of cardiac death, serious clinical complications, and delays in the receipt of effective treatments, primarily coronary reperfusion therapy. Moreover, to understand why these patients fail to react promptly to their AMI symptoms, focus group discussions and/or in-depth interviews should be carried out in patients hospitalized with AMI focusing on their levels of cognition, knowledge, and attitudes toward hospitals and health care. Additional studies are warranted in exploring the effects of educational attainment, insurance coverage, neighborhood characteristics, psychosocial aspects, and other factors that may serve as either facilitators or obstacles for patients to the timelier seeking of medical care, particularly of factors that may be amenable to modify.

### Study limitations

There are several imitations in this study. First, our data were collected from only one hospital, the VNHI in Hanoi, which limits the generalizability of our results to patients hospitalized in other medical centers across Hanoi. Some of observed patient characteristics may not reflect the general population of patients hospitalized with AMI in Hanoi; the distribution of non-STEMI appeared to be low in this study sample, though the frequency of various manifestations of AMI in Vietnamese adults is unknown. Second, due to our small sample size, we failed to identify potential factors that might relate to the prolonged delay time, information which is important for designing and implementing various educational intervention approaches to improve patient’s care seeking behavior. The subgroup analysis according to type of AMI, namely by STEMI and non-STEMI, was subject to potential bias in interpretation due to the small study sample size. Third, we were only able to calculate total duration of pre-hospital delay and we were unable to separately calculate patient delay time and extent of transportation delay since the time when patients made a treatment-seeking decision was not recorded in hospital medical records. In Vietnam, patients with signs and symptoms suggestive of AMI normally call for an ambulance or reach the hospital by themselves, and extremely few patients have a primary care doctor to consult with. In terms of transportation time, this study included only patients who reside in Hanoi. Forth, due to the retrospective design of the study, we were unable to obtain systematically collected and/or recorded information about the time of onset of symptoms suggestive of AMI in hospital medical records. This information was abstracted from notes written by different physicians, nurses, and emergency personnel which may have been collected and recorded in a non-standardized manner. Data on duration of prehospital delay were missing in approximately two thirds of Hanoi residents hospitalized with independently validated AMI, which was mainly due to missing data on the time of onset of acute symptoms suggestive of AMI, and not emergency department arrival time. Finally, we were unable to obtain information on additional patient-associated characteristics (e.g., socioeconomic status [eg., Education, occupation] and psychological factors [eg., reluctance to call family members for help or having no one around when experiencing symptoms to ask for help]) which may have impacted on patient’s medical care seeking behavior.

Our data show that patients admitted for initial AMI in Hanoi, Vietnam experienced a significant delay time in seeking medical attention after the onset of AMI suggestive symptoms. Additional full-scale data of hospitalized patients with AMI across Hanoi will be needed in order to confirm these preliminary descriptive findings. Public educational programs for the general population, specifically targeting towards women, is greatly needed to incentivize patients to seek medical assistance immediately to ensure maximum effectiveness of treatments. Further research is required to understand potential factors associated with the prolonged delay in Vietnamese patients hospitalized with AMI and the most effective ways to encourage patients to seek medical care in a timely manner.

## Data availability

The data referenced by this article are under copyright with the following copyright statement: Copyright: © 2018 Nguyen HL et al.

Data associated with the article are available under the terms of the Creative Commons Zero "No rights reserved" data waiver (CC0 1.0 Public domain dedication). 

*F1000Research*: Dataset 1. Raw dataset for Nguyen *et al.*, 2015 ‘Pre-hospital delay in Vietnamese patients hospitalized with a first acute myocardial Infarction: A short report’, 10.5256/f1000research.6943.d100892 ^23^
