# Supplementary material for: Pre-hospital delay in Vietnamese patients hospitalized with a first acute myocardial infarction: A short report
Source: F1000Res. 2018 Jan 15;4:633. Originally published 2015 Aug 27. [Version 3] doi: 10.12688/f1000research.6943.3 (PMC5790997; doi:10.12688/f1000research.6943.3)
Supplement: Peer review history

# Peer review: Referee response for version 3

**DOI:** 10.5256/f1000research.14795.r29795

**Published:** 2018-01-29

**Authors:** Polly W.C. Li

**Affiliations:** 1 The Nethersole School of Nursing, The Chinese University of Hong Kong, Shatin, Hong Kong

I have read the revised the version and wish to change the status to “approved”. My comments are addressed in the revised version.

I have read this submission. I believe that I have an appropriate level of expertise to confirm that it is of an acceptable scientific standard.

# Peer review: Referee response for version 2

**DOI:** 10.5256/f1000research.13251.r28540

**Published:** 2017-12-11

**Authors:** Polly W.C. Li

**Affiliations:** 1 The Nethersole School of Nursing, The Chinese University of Hong Kong, Shatin, Hong Kong

This report addressed an important gap in the literature about the care-seeking delay of Vietnamese in the context of acute myocardial infarction. The major limitations of the study were small sample size and retrospective nature of the study. Nevertheless, this is a good start to provide quick preliminary findings to support a more formal and large scale study to examine the phenomenon. I have several additional comments:

  

1. 69% of the sample suffered from STEMI, which is not consistent with the proportion of STEMI/NSTEMI in AMI population. This reflects that the sample is not representative and need to address in the discussion and limitation sections.
2. Retrospective medical record review is not a reliable data collection method for collecting data on pre-hospital delay. The inconsistency for healthcare professionals to determine the symptom onset time creates a significant bias to the results.
3. The very small number of NSTEMI patients made the subgroup analysis to be less appropriate.

I have read this submission. I believe that I have an appropriate level of expertise to confirm that it is of an acceptable scientific standard, however I have significant reservations, as outlined above.

# Response

**Published:** 2017-12-27

**Authors:** Hoa L. Nguyen

**Affiliations:** Institute of Population, Health and Development (PHAD), Vietnam

We would like to thank the reviewer of our article for the helpful comments. We have attempted to address each of the concerns raised about our submission and have indicated in the revised text where changes have been made. We believe that the manuscript is considerably stronger than before.

1. 69% of the sample suffered from STEMI, which is not consistent with the proportion of STEMI/NSTEMI in AMI population. This reflects that the sample is not representative and need to address in the discussion and limitation sections.

*Response: We have acknowledged this limitation in the revised discussion (Study limitations section), though we are unsure what the distribution of type of AMI may be in the Vietnamese population. In our conclusions section, we have emphasized the need for full-scale surveillance of Hanoi residents hospitalized with AMI at all Hanoi hospitals to confirm these preliminary descriptive findings.*

 2. Retrospective medical record review is not a reliable data collection method for collecting data on pre-hospital delay. The inconsistency for healthcare professionals to determine the symptom onset time creates a significant bias to the results.

*Response: Due to the retrospective nature of this observational study, there was no systematic method established to record the time of onset of symptoms suggestive of AMI (e.g., chest pain) in a standardized manner by trained health professionals. While we would have preferred to have directly interviewed hospitalized patients with AMI in a standardized manner, as noted in response to a similar query by the first and second reviewers, this information was abstracted from notes written by different physicians and nurses and* *emergency personnel which may have been collected and recorded in a non-standardized manner* *.* *We have noted this limitation in the discussion section of the revised manuscript (* *Study limitations section* *)* *.*

3. The very small number of NSTEMI patients made the subgroup analysis to be less appropriate.

*Response: We agree with the reviewer and have acknowledged this limitation in the revised discussion (Study limitations section). However, in this brief report, we would prefer to describe the extent of prehospital delay for patients with a STEMI and NSTEMI separately given the extremely limited data presently available on this topic in Vietnam and due to differences in the natural history of these conditions. In our conclusions, we have emphasized the need for full-scale surveillance of Hanoi residents hospitalized with AMI at all Hanoi hospitals to confirm these preliminary descriptive findings.*

# Peer review: Referee response for version 2

**DOI:** 10.5256/f1000research.13251.r24711

**Published:** 2017-09-13

**Authors:** Dao Thi Mihn An

**Affiliations:** 1 Department of Epidemiology, Institute for Preventive Medicine and Public Health, Hanoi Medical University, Hano, Vietnam

I have read the Summary of revisions presented by the author of the paper "Pre-hospital delay in Vietnamese patients hospitalized with a first acute myocardial infarction: A short report". Generally, the author has improved the weak points indicated by reviewers by adding information, discussing the limitation issues, or re-structuring within each section of the paper.

 The main finding of this paper supplies evidence of pre-hospital delay among AMI patients in Vietnam that would probably result in further comprehensive study of this issue in the near future in Vietnam.

 I agree with the revisions of the author, and approve this paper.

I have read this submission. I believe that I have an appropriate level of expertise to confirm that it is of an acceptable scientific standard.

# Peer review: Referee response for version 1

**DOI:** 10.5256/f1000research.7478.r15408

**Published:** 2016-08-03

**Authors:** Dao Thi Mihn An

**Affiliations:** 1 Department of Epidemiology, Institute for Preventive Medicine and Public Health, Hanoi Medical University, Hano, Vietnam

There were not any official publications in Vietnam regarding issue of pre-hospital delay in Vietnamese patients hospitalized with a first acute myocardial infarction until now. Therefore, the short report on this issue written by Hoa *et al* is highly appreciated.

 This study was conducted with a small and convenient sample of 103 AMI patients with their first AMI and the study approach was to review AMI patients' medical records after their discharge that must be embedded biases as it was a retrospective study. The inconsistence of asking and identifying time of delay by different health workers  at the time of hospitalization and the different subjects who supplied information for health workers should be considered the main problems of the validity of data in this study beside 3 other limitations that the author has discussed on this paper. Moreover, it was likely that there were missing value regarding delay time occurred during process of extracting data from AMI patients' medical record.

 However, this study initially indicated  2 very important findings that pre-hospital delay time for hospitalization among AMI patients was much longer than that of AMI patients in the local and worldwide and patients who had a delay time over 6 hours were more likely to be female compared to -their counterparts. These findings make help in giving suggestion for further full-scale data of hospitalized patients with AMI across Hanoi in order to confirm these initial findings.

 I would like very much to approve for this short report as it is the starting point for further large-scale study in this issue in Vietnam where such information are still rare.

I have read this submission. I believe that I have an appropriate level of expertise to confirm that it is of an acceptable scientific standard, however I have significant reservations, as outlined above.

# Response

**Published:** 2017-07-24

**Authors:** Hoa L. Nguyen

**Affiliations:** Institute of Population, Health and Development (PHAD), Vietnam

*Response: Due to the retrospective nature of this observational study, there was no systematic method established to record the time of onset of symptoms suggestive of AMI (e.g., chest pain) in a standardized manner by trained health professionals. As noted in response to a similar query by the other reviewer, this information was abstracted from notes written by physicians and nurses and* *emergency personnel which may have been collected and recorded in a non-standardized manner. Duration of prehospital delay was missing in approximately two thirds of Hanoi residents hospitalized with AMI in 2010, which was mainly due to missing data on the* *time of onset of symptoms suggestive of AMI.* *We have noted this limitation in the discussion section of the revised manuscript* *.*

# Peer review: Referee response for version 1

**DOI:** 10.5256/f1000research.7478.r10208

**Published:** 2015-10-13

**Authors:** Felix Alvaro Medina Palomino

**Affiliations:** 1 Alberto Hurtado School of Medicine, Cayetano Heredia Peruvian University, Lima, Peru

1. It is not clear how the author obtained their samples.
2. There is a very small population to study and to obtain valid conclusions from.
3. It is uncertain how the time of chest pain was recorded. It appears there is no system that allows for a correct determination of the precise moment the patient arrives at the ER, due to the fact that in many patients this particular data is missing.
4. The report is based on more than a hundred patients but in fact the author only presents data from 65 patients.
5. There is no explanation about the findings nor factors.
6. Because only a few patients were enrolled, it is not possible to determine factors related to early or delayed arrival at the hospital.
7. Finally some data, which are in fact results, are written in the methods.

I have read this submission. I believe that I have an appropriate level of expertise to state that I do not consider it to be of an acceptable scientific standard, for reasons outlined above.

# Response

**Published:** 2017-07-24

**Authors:** Hoa L. Nguyen

**Affiliations:** Institute of Population, Health and Development (PHAD), Vietnam

1. It is not clear how the author obtained their samples.

*Response: We have described the study population in greater detail in the Case Ascertainment section of the Methods (page 3, para 2). In brief, lists of* *patients discharged from the Vietnam National Heart Institute in 2010 with possible AMI were obtained and International Classification of Disease-10 codes for possible AMI (I20-I25) were reviewed. The diagnosis of AMI was made on the basis of criteria developed by the World Health Organization which includes a suggestive clinical history, serum enzyme elevations above the hospital’s normal range, and serial electrocardiographic (ECG) findings during hospitalization consistent with the presence of AMI; at least 2 of these 3 criteria needed to be present for an AMI to have occurred.*

*We restricted our patient population to those living in the city of Hanoi and with information on prehospital delay recorded in hospital medical records. We have added this information in the revised manuscript.* 

       2. There is a very small population to study and to obtain valid conclusions from.

*Response: We have acknowledged this limitation in the revised discussion. In our conclusions, we have emphasized the need for full-scale surveillance of Hanoi residents hospitalized with AMI at all Hanoi hospitals to confirm these preliminary descriptive findings.*

     3. It is uncertain how the time of chest pain was recorded. It appears there is no system that allows for a correct determination of the precise moment the patient arrives at the ER, due to the fact that in many patients this particular data is missing.

*Response: Due to the design of this retrospective study, whose information was based exclusively on information contained in hospital medical records, there was no systematic method to collect and record the time of onset of symptoms suggestive of AMI (e.g., chest pain) since  these data were collected and recorded in hospital records by nurses and physicians with varying degrees of expertise and interest in collecting this information. Data on* *duration of prehospital delay were missing in approximately two thirds of Hanoi residents hospitalized with AMI in 2010, which was mainly due to missing data on the* *time of onset of acute symptoms suggestive of AMI, and not ER arrival time* *. We have included this limitation in the discussion section of the revised manuscript* *.*

**

     4. The report is based on more than a hundred patients but in fact the author only presents data from 65 patients.

*Response: The present report is based on 103 patients with independently confirmed AMI. Data on duration of prehospital delay (continuous outcome) were  available in only 65 patients, whereas the other 38 patients had data available only for  a time interval (categorical outcome). Inasmuch, we have analyzed duration of prehospital delay as a continuous outcome in 65 patients and as a categorical outcome in 103 patients. We have modified the results section to reflect our approaches to the analysis of these data in the revised manuscript.*

     

      5. There is no explanation about the findings nor factors.

*Response: In this brief report, our primary goal was to present descriptive data about the extent of prehospital delay in patients hospitalized with AMI at the major teaching hospital in Hanoi, information which is unknown and sorely lacking in Vietnam. Since the sample size is small, we were unable to examine factors associated with prolonged delay, information which is   important for designing and implementing various educational intervention approaches to improve patient’s care seeking behavior* *.* *We have emphasized this limitation and the need for future studies in the revised manuscript.*

       6. Because only a few patients were enrolled, it is not possible to determine factors related to early or delayed arrival at the hospital.

*Response: Please see the above response (#5).*

      7. Finally some data, which are in fact results, are written in the methods.

*Response: We have removed data on prehospital delay in the method sections and added them into the results section of the revised manuscript.*
